# Miniaturized Monitors for Assessment of Exposure to Air Pollutants: A Review

**DOI:** 10.3390/ijerph14080909

**Published:** 2017-08-12

**Authors:** Francesca Borghi, Andrea Spinazzè, Sabrina Rovelli, Davide Campagnolo, Luca Del Buono, Andrea Cattaneo, Domenico M. Cavallo

**Affiliations:** Dipartimento di Scienza e Alta Tecnologia, Università degli Studi dell’Insubria, Via Valleggio 11, 22100 Como, Italy; sabrina.rovelli@uninsubria.it (S.R.); davide.campagnolo@uninsubria.it (D.C.); luca.delbuono@uninsubria.it (L.D.B.); andrea.cattaneo@uninsubria.it (A.C.); domenico.cavallo@uninsubria.it (D.M.C.)

**Keywords:** air pollution monitoring, citizen science, exposure assessment, global positioning system (GPS), participatory sensing, particulate matter, portable monitoring, wireless sensor network

## Abstract

Air quality has a huge impact on different aspects of life quality, and for this reason, air quality monitoring is required by national and international regulations. Technical and procedural limitations of traditional fixed-site stations for monitoring or sampling of air pollutants are also well-known. Recently, a different type of miniaturized monitors has been developed. These monitors, due to their characteristics (e.g., low cost, small size, high portability) are becoming increasingly important for individual exposure assessment, especially since this kind of instrument can provide measurements at high spatial and temporal resolution, which is a notable advantage when approaching assessment of exposure to environmental contaminants. The aim of this study is indeed to provide information regarding current knowledge regarding the use of miniaturized air pollutant sensors. A systematic review was performed to identify original articles: a literature search was carried out using an appropriate query for the search of papers across three different databases, and the papers were selected using inclusion/exclusion criteria. The reviewed articles showed that miniaturized sensors are particularly versatile and could be applied in studies with different experimental designs, helping to provide a significant enhancement to exposure assessment, even though studies regarding their performance are still sparse.

## 1. Introduction

Air pollution may result in huge impacts, causing different effects on human health, on the environment (e.g., ecosystem damage) and on the economy of industrialized and developing countries [1,2,3]. For these reasons, air quality monitoring is typically required by national and international regulations to assess systematically the environmental exposure of the general population to multiple environmental contaminants [3,4].

The equipment used to meet international standards regarding air quality/air pollution measurement is, at present, characterized by high cost and a high level of maintenance [3,5]. For example, the purchase and installation of single gas-analyzer can cost between £10,000 and £15,000 while the purchase and installation of particulate monitoring devices in existing station can cost between £10,000 to £25,000. Finally, the purchase of a multi-pollutant analyzer can cost £50,000–£80,000 [6,7]. In addition to the economic issue, traditional air quality stations are placed at strategic fixed-site locations and can provide accurate data only for a restricted area. This is a disadvantage because measurements at adequate spatial scales are essential for monitoring air pollution in heterogeneous environments such as the urban environment, characterized by different emission sources [6,8,9,10]. High spatial (and temporal) resolution data are especially important in the air pollution field, due to the highly location-dependent concentration of atmospheric pollutants, particularly in the urban environment, which is characterized by high variability in terms of point pollutant concentrations [11]. Other disadvantages related to the use of fixed-site monitoring stations are related to the necessity of other support infrastructures (e.g., secure enclosures, power supply, etc.) [6,11]), of a dedicated area, and the need for maintenance and of continuous power consumption [12,13]. However, since traditional stationary sampling devices are usually expensive and complex to use, the development of portable sensors for the measurement of airborne pollutant concentrations has provided data with high temporal resolution characterized by a real-time response [14]. These portable instruments have been widely used in several studies, significantly improving the assessment of human exposure to atmospheric pollutants, since these instruments are able to measure exposure at an individual level (defined as the exposure constantly measured in proximity—within 3 m—to the subjects) or at personal level (which is preferable for assessing human exposure, as it is representative of the contaminant concentration in the breathing zone) [15,16].

Moreover, personal monitors can provide air pollutant exposure concentrations for specific and selected subject categories (e.g., susceptible individuals, workers, etc.) and, furthermore, can be used both indoors and outdoors. Several in-field campaigns have been conducted in the last years, using portable measurement devices, to evaluate the individual or personal exposure to different air pollutants and across different scenarios [17,18,19,20,21,22,23]. At present, the main limitation of these monitors is that portable devices are generally characterized by worse performance in terms of accuracy than the commonly used standard techniques [24]. Therefore, in summary, the quality of future exposure assessment studies depends strongly on the improvement of direct-reading portable sensors for airborne pollutant measurements, in terms of their compactness, portability, reliability, accuracy, and costs [25]. In recent years, a new kind of extremely portable air pollutant monitor has been developed [26,27]; these sensors are generally manufactured using micro-fabrication techniques and contain micro-electro-mechanical systems (made of microfluidic, optical and nanostructured elements), allowing them to be compact, lightweight, inexpensive, and energy-efficient, with extremely low-power consumption. These devices are usually also completed by advanced computing power for data handling and by software packages for data elaboration and visualization [28]. Then, miniaturized sensors devices are usually low cost (i.e., ~£100–£5000), easy to use and easily portable, and they can provide data with high spatial and temporal resolution [29,30] and real-time continuous measurement of air pollutants [26]. These devices are continuously being improved, and their use is becoming increasingly widespread [29,30,31,32,33] because of the several previously described advantages. Considering the growing use of these miniaturized air pollutant monitors, the aim of this review is to identify and report studies that have used portable airborne gas and particulate matter (PM) sensors and the new integrated technologies such as Global Positioning System (GPS), wireless communication module and web/smartphone applications and to present the principal results provided by these studies. Although a recent paper [34] reported the characteristics and applications of portable gaseous air pollution monitors, there are no systematic review regarding the use on field of miniaturized PM and gas sensors and regarding the benefits arising the use of integrated technologies abovementioned. 

The paper identification process used for the systematic review of the literature is described in Section 2. The results reported by article selection and screening are illustrated in the initial part of Section 3 while in the second part of this section are presented the new technologies to support individual and personal monitoring (GPS; wireless communication module, web or smartphone application). Principal results show how miniaturized monitors are continuously improved and how their use is becoming increasingly widespread, having the potential to improve human exposure assessment studies. 

## 2. Materials and Methods 

We conducted a systematic review using outcomes in three different databases (ISI Web of Knowledge, PubMed and Scopus). For each database, we used a list of keywords, which was the same for the three databases (Table 1). The keywords and the query structure were arranged as a function of the writing rules required by the selected database. 

**Figure 1:**
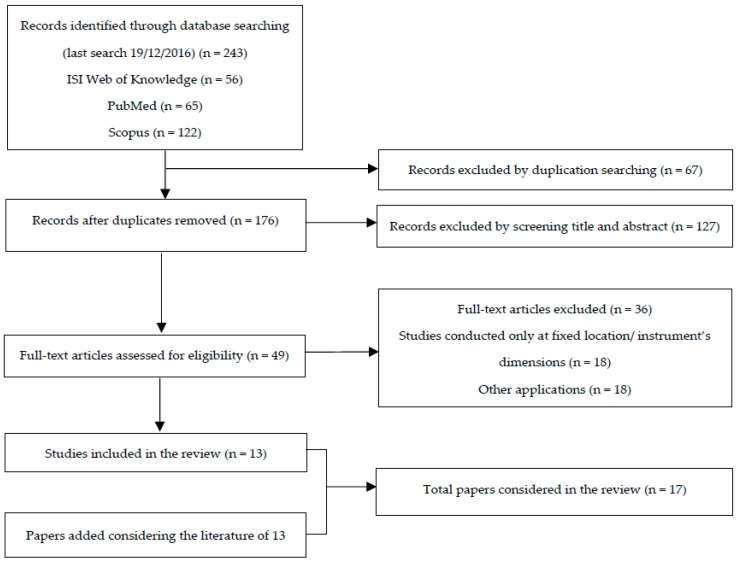
Flowchart of literature searched and reviewed (modified from Moher et al. [35]).

We found a total of 56 papers in ISI Web of Knowledge, 65 papers in PubMed and 122 papers in Scopus (last search: 19/12/2016). Papers were detected and then selected following chosen inclusion and exclusion criteria. First, we decided not to use time filters regarding year of publications and to consider only scientific papers written in the English language. For this reason, data concerning conference proceedings were not reported. Other exclusion criteria considered concerned the kind of the study: we decided to consider only studies conducted in the field with mobile monitors (e.g., mobile monitors used in fixed locations were not considered). Moreover, only “miniaturized” sensors (i.e., with the greater dimension smaller than 20 cm) were selected. Notably, the proposed dimensional criteria were not confirmed in the scientific literature, but it was an arbitrary subdivision with a certain level of subjective decisions. Unfortunately, it was not possible to make a selection according to the price of the sensor because papers did not report costs and because several instruments were developed by universities so prices would not be available in any case. Finally, since several miniaturized sensors able to measure different environmental parameters (e.g., temperature, relative humidity, noise, pollutants) are available on the market, we considered only papers concerning environmental pollutants, with no other restrictions about kind of pollutant. After a selection in accordance with the aforementioned inclusion/exclusion criteria, only 13 papers were found to be suitable for the present review. Therefore, to provide an overview as accurate as possible about studies that used miniaturized monitors in the field, we decided to extend the number of papers considering also those articles reported as references in the 13 selected studies. The reported literature was consequently analyzed following the same inclusion/exclusion criteria. Four papers were added in this way, and a total of 17 papers was finally reported in the present review A. flowchart of the literature research and review process (modified from Moher et al [35]) is reported in Figure 1. 

## 3. Results and Discussion

The next paragraphs report studies relative to campaigns in the field that used miniaturized sensors for PM and gas in air quality monitoring campaign and instrument validation. We also report technical and instrumental innovations (GPS, wireless communication and web/smartphone applications), the use of which is becoming increasingly common. As evidence, the scientific community is increasingly attentive to these topics and the number of scientific studies regarding these matters is rising. 

The number of papers analyzed in this work, total raw results and selection by publication year is reported in Table 2. There is a positive trend concerning the number of articles per publication year, which could be interpreted as an increased scientific interest in the topic.

### 3.1. Particulate Matter Sensors

The present subsection is focused on studies regarding particulate matter (PM) sensors [3,5,31,36,37]. Sensor characteristics are reported in Table 3, whereas summary information about the experimental design are reported in the supplemental material (Tables S1 and S2).

Regarding the experimental design of the selected studies, miniaturized sensors were often placed on public and private transport devices such as vehicles, bicycles, and public transportation, but only for a limited amount of time, probably due to the battery life problems, commonly related to PM sensors. In such cases, the use of a secondary power supply with customized voltage regulation (and/or supplemental batteries) can contribute to extend the duration of monitoring, even if this can significantly affect portability of the instruments. Velasco et al. [3] conducted a study focused on the use of a high-portability mobile sensor network system, with easy data acquisition and maintenance. The sensor network included commercial PM_10_ and O_3_ sensors. In this study, different tests were carried out in various locations: tests were performed in controlled environments (indoor/outdoor locations) and during different field campaigns (e.g., urban/rural locations) in mobile and static tests. The results arising from mobile tests (the system was mounted on a bicycle) showed that measurements did not suffer significant variation compared to the measurements made with instruments used as fixed monitors. Additionally, during long-run mobility tests, the prolonged use of the device did not reveal relevant problems. In conclusion, the authors asserted that measurements carried out with the use of a mobile sensor network are less accurate than reference instruments used at fixed locations by the Regional Environmental Agency. However, this kind of sensor has the potential to provide insight about air pollution and to complement data acquired from official and reference monitoring stations. The authors also reported and highlighted how mobile wireless systems may be able to improve spatial and temporal resolution, thus improving exposure assessment studies. Other authors [37] conducted field tests in different environments, measuring PM_2.5_ concentration, both indoors and outdoors, on a bus journey and during walking. The results arising from field tests showed that the sensor provided a good performance for immediate measurement in living environments. Moreover, a comparison between instruments used and the reference instruments used at fixed locations by the Hong Kong Environmental Protection Department showed that PM_2.5_ data were characterized by a reasonable accuracy. As a further result, the authors considered that an extensive use of this kind of sensor could contribute to raising the public awareness regarding air quality in microenvironments. Another way to assess the human exposure to air pollutants is through the use and interpretation of pollutant maps or models, which can also be developed on the basis of concentration results obtained by miniaturized sensors. For example, Hasenfratz et al. and Mueller et al. [31,36] reported results from a two-year campaign conducted in Zurich, measuring ultrafine particles (UFP), O_3_, CO and NO_2_. In this campaign, a land-use regression model was developed to create maps of air pollutant concentrations with high temporal and spatial resolution, monitoring the pollutant concentrations using 10 sensor nodes installed on top of public transport vehicles. The results showed that despite the accuracy of the obtained maps being influenced by the relatively small number of measurements, these maps could be useful for a detailed exposure assessment because of their high spatial and temporal resolution. Finally, [5] described an innovative approach for the integration of physical and digital worlds through aggregation of Internet of Things (IoT), demonstrating how IoT and Augmented Reality (AR) could provide a new way for sharing data. Additionally, in this case, the authors thought that this new approach would promote environmental issues, increasing interaction between general population and environmental data.

### 3.2. Gas Sensors

A larger number of papers concerning gas sensor monitoring were found. Sensor characteristics are reported in Table 3, while a study summary is reported in the supplemental material (Tables S1 and S2). Most of the selected papers were related to the monitoring of CO [5,6,9,29,31,36,38,39], CO_2_ [5,9,29,40,41,42,43,44], O_3_ [3,5,9,29,31,36] and NO_2_ [6,9,29,31,38], even though other pollutants were sometimes considered, such as NO [5,6,29], SO_2_ [29,38]; VOC [45], and hydrocarbons and acids [46]. Several studies reported results regarding wearable sensors provided to pedestrians. For example, Chen et al. [45] presented a wearable VOC sensor able to provide information about indoor and outdoor concentrations of selected pollutants. In this study, the sensor was validated under real-world conditions and across different scenarios (i.e., different works and applications). The results regarding field tests demonstrated the goodness of data obtained and the ability of this kind of sensor to greatly improve knowledge of personal exposure to environmental contaminants. In particular, the VOC sensor performance was validated using gas chromatography and selected ion flow tube mass spectrometry as reference methods, showing accuracy higher than 81%. Moreover, a comparison conducted outdoors showed results with accuracy values higher than 84%, demonstrating the capability of the tested sensor to provide reliable measurements in outdoor environments. Similar results concerning the development and testing of wearable devices for hydrocarbons and acids were reported by Negi et al. [46]. Additionally, in this case, portable monitors provided accurate and real-time measurements. The validation of sensor performance was carried out in different scenarios, involving operators from different working fields and using GC/MS (gas chromatography/mass spectrometry as a reference method. The authors reported comparison data characterized by a high degree of correlation and with a relative error of 2% (r^2^ = 0.99). The authors also highlighted that wearable sensors can be used for remotely detecting the risk of potential toxic exposure, helping to better understand the nature of the exposure. The potential of miniaturized sensor networks in the urban environment and their ability to provide data at an adequate scale has also been highlighted by Mead et al. [6]. In this study, the authors performed measurements (concerning NO, NO_2_ and CO concentrations) via portable devices (held by pedestrians and cyclists) and via static stations, across different urban environments. Moreover, laboratory tests and validations were carried out. The authors remarked first the inability of fixed stations to fully characterize the urban environment and the necessity to use environmental networks characterized by high spatial and temporal resolution in this kind of heterogeneous environment. Second, Mead and collaborators showed that air quality sensor networks are now feasible for widespread use for monitoring at an environmental level, complementing other measurement methods. Other authors [42,44] conducted analogous monitoring campaigns and field tests, reporting similar results. Personal and continuous CO_2_ monitoring was used by Gall et al. [42] in both indoor and outdoor campaigns, to understand levels and influencing factors in personal exposure to the selected pollutant. Kanjo et al. [44] provided an evaluation of the CO sensor in a school environment, demonstrating the feasibility of an extensive environmental monitoring with the use of mobile sensing devices. 

As for PM sensors, Several studies considered the use of gas sensors on private or public transports. For example, Lo Re et al., Al-Ali et al. and Guevara et al. [9,38,39] placed environmental sensors on public transport and reported similar results, namely, that mobile monitors were characterized by good performance. The authors also highlighted how vehicular sensor networks must be considered as an innovative approach to environmental monitors. In addition to the public transport support, other kinds of transport have been used in reported studies. In a study conducted by Eisenman and collaborators [40] monitors were presented and tested during bicycle trips on different routes while test and field campaigns with positioning of monitors on different transports were reported by different authors [29,41,43]. Other studies, already reported in the previous paragraph referring to particulate matter sensors, refer also to gas monitoring [3,5,31,36] and, for this reason, they have not been reported in this paragraph.

### 3.3. Accessability of Data

At present, the way to communicate and share scientific data is changing. In the opinion of the authors, three implementations in personal exposure assessment are particularly interesting and often reported in selected studies: (i) Integration of personal monitoring with Global Positioning System (GPS); (ii) Communication and data transfer via wireless; (iii) Data communication via web or smartphone applications.

The simultaneous use of these implementations could help the citizen-scientist to have more awareness about atmospheric pollution, to share data and to try to mitigate their exposure conditions, raising community awareness about air quality [5,39]. Furthermore, sensors coupled with an efficient delivery of sensed information could provide benefits to society, improving emergency response [39]. Moreover, the simultaneous use of miniaturized sensors and, in particular, GPS can become a great support to different and innovative studies (e.g., innovative approach in the epidemiological research). These new and innovative support elements (wireless, GPS and web/smartphone applications) and their important contribution in exposure assessment studies will be further discussed below. A first and general overview of their use in the considered papers is reported in Table 4. 

At present, geo-referencing of data is becoming increasingly important, due to the possibility of understanding pollution patterns and pollution hotspots. Furthermore, the use of geo-referred data may define the human exposure more accurately. Fifteen articles out of the total 17 studies considered in the present review (equal to 88% of the total) used a GPS in the study protocol. Different kinds of GPS have been used, both connected to a mobile phone or as separate instruments. Characteristics (e.g., acquisition/navigation/tracking sensitivity, hot and cold start time, positional accuracy error and speed accuracy) may obviously change, and even provided data can be different [3]. Clearly, the fundamental data are related to the position (normally given as longitude and latitude), but other information can be supplied such as data validity checksum, velocity, heading, date, magnetic variation and direction, mode and checksum [38]. Other than coordinates, other kinds of information can be derived from GPS system results. For example, [41] used an activity classification model to determine the transportation mode (e.g., staying position, walking, driving). In this case, the system used results from activity classification as inputs to the emission factor model, to generate estimates regarding human exposure. In several studies, the main problem encountered in the use of GPS was closely related to the number and position of GPSs and related satellites. Position accuracy could not be very good since the GPS signal could be blocked by buildings or when the GPS signal results were totally blocked due to the overhead cover [37]. GPS also seems not always to be functional in some common environments such as among tall buildings or under dense overgrowth (tree canopy) [40].

Regarding communication and data transfer via wireless, 14 articles out of 17 (82% of the total) and 10 articles of 17 (59% of the total) were endowed, respectively, with wireless communication mode and smartphone/web application. Few studies used smartphone connected to pollutant sensors to collect data, but this innovative approach could be very useful. Kanjo et al. [44] highlighted three advantages of using a mobile phone: (i) mobile phones are carried around by a large percentage of population; (ii) the mobile phones can be used to process, store and transfer other kinds of data (such as photos and messages), and (iii) collection using mobile phones should be more energy efficient, because data are sent directly to the phone bypassing the entire sensor network. Moreover, a wireless network may be created, with the aim of providing real-time information about environmental and human health hazards [45] and, using smartphone and associated wireless technology, data can be transferred and shared more easily, and exposure data can be obtained remotely [46]. Furthermore, several authors highlighted how Wireless Sensor Networks (WSNs) can simplify the use of sensors. WSNs can eliminate barriers related to installation, remove connectors and increase scalability. Guevara et al. [39] showed typical obstacles and barriers in an Intelligent Vehicle Network (IVN) system: bundles of lead wires are subject to breakage, and they represent a significant installation and long-term maintenance cost. Further problems reported by authors referred to the scalability of the sensors, which is limited by vendor-specific protocols. Analysis of selected papers shows that different kinds of communication protocols can be used for personal monitoring purposes. Standards such as 802.15.1 (Bluetooth), 802.15.4 (ZigBee), 802.11 (Wi-Fi), 3G/GPRS, GSM/GPRS, Bluetooth Low Energy (BLE), standard IEEE 1451 family, 802.15.4 (Intra-BAN) and Radio Frequency (RF) communication are commonly adopted as viable wireless interfaces [3,37,39]. However, most of the selected studies adopted the Bluetooth module for wireless communications, probably because Bluetooth has been recognized as an effective mode for short range data communication due to its relatively low power consumption and low-cost [37]. Obviously, a wireless communication module is necessary for the development and the use of web or smartphone applications, due to the type of real-time data. Data returned by the web or smartphone applications are of different kinds: normally the applications characterized by a user-friendly interface show data concerning date, time, pollutant monitoring, position and concentration results. Different applications also have a notification service that warns if the environmental readings exceed threshold values. Data summary, elaborations, precautionary measures to adopt and download functions are also commonly provided by the system. Often, applications are also able to provide data concerning the status of the monitor (e.g., pump, valves and battery life) while in other applications, users can select different application scenarios (e.g., industrial solvent, motor vehicle emission).

### 3.4. Impact on the Assessment of Human Exposure

We observed that both traditional and new miniaturized monitoring devices present advantages, especially related to the capability of providing continuous and real-time data characterized by high spatial and temporal resolution. Traditional fixed-site monitoring stations provide data characterized by good quality but, due to high costs and several position problems, the number of these stations is reduced and, consequently, data provided lacks an adequate spatial coverage. New miniaturized and low-cost devices generally have worse data quality (for the time being), but they are able, also thanks to the new paradigm of monitoring “citizen-science”, to have higher spatial and temporal resolution, which is fundamental in complex environments such as urban environments. The aim of this review was not to evaluate the performance of miniaturized sensors, even if, after a preliminary analysis, they seemed to be less accurate as compared with reference methods. Moreover, a comparison between sensor performance was not possible because the considered studies used different sensors and sampling protocols, across various scenarios and, furthermore, only a few papers reported data concerning the comparison between miniaturized sensors and reference methods. For this reason, the evaluation of miniaturized sensor performances should be considered in future specific studies, to establish appropriate methods for evaluation and validation, as well as to provide adequate operating procedures to ensure the obtaining of accurate data. Moreover, performance and reliability of these sensors have yet to be fully evaluated, especially for long-term measurements [26]. Particularly, performance in areas with poor air quality, cross interferences and influences of temperature and relative humidity should be evaluated [26,47]. In this regard, the intercomparison and the performance evaluation (especially as regards the field performance) of miniaturized sensors is necessary, because if sensor performance results are to be validated (e.g., via comparison with reference methods), these miniaturized sensors could be used as support to fixed air quality monitoring networks to achieve a broader spatial coverage, to provide a more representative characterization of exposure [48]. However, despite this possible lack in their accuracy, and due to the abovementioned advantages, miniaturized monitors are becoming increasingly important in community and individual exposure assessment studies and can potentially be used in different application, such as outdoor/indoor air pollution monitoring and community/individual exposure [26]. In this review, we also wanted to emphasize the increasingly innovative role of miniaturized pollutant sensors and their different applications. We first found how, at present, different miniaturized sensing devices are available on the market, and how improvements in sensor technologies are currently emerging. Overall, there is a need to develop personal environmental monitoring systems, especially integrating measurement devices, mobile application on smartphones and GPS data [37]. Second, we found how miniaturized sensors could support or become a new way for human exposure assessment, especially due to their capability to measure air pollutant concentrations at adequate spatial and temporal scale and to their great versatility (such as the ability to adapt to different experimental designs). Reviewed papers reported that measurements conducted at high spatial and temporal resolution could contribute to scientific understanding and address economic, policy and regulatory issues, as well as air quality and human exposure [6]. All these positive features may lead to the effective use of these sensors in different environmental and human health fields. Moreover, when sensors are used by the general population, they can be useful to report data relative to an immediate surrounding or to a selected location, and this knowledge will help citizens make the decision regarding quality of life [29]. In particular, if connected in networks, these sensors can provide data representative of high spatial and temporal variability in pollutant concentrations for a wider area, unlike traditional monitoring stations [49]. Moreover, Micro Sensing Units (MSUs) can gather high spatial and temporal resolution data from numerous nodes [2], especially if they are connected by a Wireless Distribute Environmental Sensor Network (WDESN).

An example of this use is the “Citizen Science”. Citizen-scientists are described as citizens involved in collecting, categorizing, transcribing or analyzing scientific data [26,47,50]. Citizen-science is presently considered an important implementation to scientific studies, especially because it can increase the spatial coverage and time resolution data, also thanks to the increasing use of personal devices such as smartphones [1]. According to the “Participatory Sensing” (PS) definition, users can acquire and make available to other people data of interest, such as data regarding air quality, pollutant concentrations (e.g., environmental data, weather and traffic information, also related to geographical position and time of data collection, intelligent transportation and route planning) [12,13,51]. Data are subsequently reported to a central server through wireless communication. Data analyzed and processed by the server are presented and displayed on participant smartphones in different forms (e.g., graphical representation or maps) [12]. This innovative approach might be able to satisfy the increasing interest in mobile air quality sensor network applications, as noticed by different studies [4] and provide increased coverage of monitored areas (in time and space), in addition to facilitating learning and increasing citizen awareness of environmental issues [10]. 

In addition, the importance of Internet in the environmental research field should be emphasized. Traditional dissemination channels such as television and radio contribute to a common sense but often do not provide update data, and even data are not directly accessible by users [32]. With progress in mobile, miniaturized and on-line technologies, more environmental data can be spread and characterized by higher spatial and temporal resolution. In this way, users can receive personalized information (in terms of time, area of interest and kind of environmental data request). At present, several initiatives promote tests of environmental sensor devices that are coupled with smartphones and that can connect to web portals [52]. Then, principal pro and cons of Miniaturized Monitors (MMs) which resulted from this review are reported in Table 5.

Finally, we have chosen not to report data relative to pollutant sensors used at fixed stations, but it is important to remember their importance in air quality measurement and exposure assessment, especially if they are connected in a network of sensors. As reported by Castell et al., 2015 [29], the combination of mobile sensors and fixed stations might be able to foster the development of spatial models, helping to create a new approach to the human exposure assessment. Finally, we found three different innovations that have the potential to significantly improve human exposure assessment studies. These innovations are related to the integration of GPS, wireless communication mode and smartphone or web applications. 

## 4. Conclusions

Due to their characteristics, miniaturized sensors for the measurements of airborne gas and PM could provide a significant enhancement in exposure assessment studies, increasing the spatial and temporal resolution of human exposure data and incrementing the awareness and the data-sharing process. The articles that were reviewed also showed that miniaturized sensors are particularly versatile and could be applied in studies with different experimental design, helping to ensure high quality and in high-sensitivity exposure assessments (particularly in participatory and ubiquitous monitoring campaigns), even though studies regarding their accuracy or the comparison between miniaturized sensors and reference methods still seem to be sparse. 

## Figures and Tables

**Table 1:** Query used for the search in three different databases: ISI Web of Knowledge, PubMed and Scopus.

Database	Search Query
ISI Web of Knowledge	(TS = (“air quality”)) and (TS = (“sensor network” or “wearable sens*” or “crowd sensing” or “participatory sensing” or “mobile sensor node” or “low cost sensor” or “citizen science”))
PubMed	(air quality) and ((sensor network) or (wearable sens*) or (crowd sensing) or (participatory sensing) or (mobile sensor node) or (low cost sensor) or (citizen science))
Scopus	(TITLE-ABS-KEY (“air quality”)) and (TITLE-ABS-KEY (“sensor network” or “wearable sens*” or “crowd sensing” or “participatory sensing” or “mobile sensor node” or “low cost sensor” or “citizen science”))

**Table 2:** Number of papers analyzed per publication year. The sum of all papers resulting from raw research within ISI Web of Knowledge, PubMed and Scopus (inclusion/exclusion criteria not considered) were reported in the second column. The number of papers selected and present in this work was reported in the third column. The only paper published before 2004 is reported in brackets.

Publication Year	Sum of Papers	Papers Considered in This Review
(1977)	1	0
2004	1	0
2005	0	0
2006	5	0
2007	0	0
2008	12	1
2009	6	1
2010	11	1
2011	12	2
2012	17	3
2013	26	0
2014	39	3
2015	57	3
2016	56	3

**Table 3:** Particulate and gas sensor characteristics as reported in the selected papers. Data that were not directly acquired from the paper are reported in italics. In the case of missing data within the reference papers, data were acquired from the literature cited in bibliography or from external sources (retailer’s site).

Reference	Study	Pollutant	Sensor/Instrument	Dimensions and Weight	Measuring Principle	Operational Range	Sensitivity
**PM Sensors**							
[37]	Wong et al., 2014	PM_2.5_	GP2Y1010AU0F (Sharp)	89 × 113 mm	Light scattering	n.a	n.a
**Gas Sensors**							
[38]	Al- Ali et al., 2010	CO	All sensors: Alphasense	n.a	All senors: electrochemical	CO: 0–1000 ppm	CO: <1.5 ppm
		NO_2_				NO_2_: 0–20 ppm	NO_2_: 0.02 ppm
		SO_2_				SO_2_: 0–20 ppm	SO_2_: <0.1 ppm
[29]	Castell et al., 2015	O_3_	All sensors: Alphasense (Series B)	*All sensors: 32 mm (sensor diameter)*	All sensors: electrochemical	All sensors: concentration typically found in urban environment	n.a
		CO					
		CO_2_					
		NO					
		NO_2_					
		SO_2_					
[45]	Chen et al., 2012	VOCs	n.a	Not much larger than common smartphone (<300 g)	n.a	4 ppb–1000 ppb	Resolution < 4 ppb
[40]	Eisenman et al., 2009	CO_2_	7001 CO_2_/Temperature monitor (Telaire)	*150 × 70 mm*	*Absorption Infrared*	*0–2500 ppm or 0–4000 ppm*	*±1 ppm*
[41]	Fu et al., 2012	CO_2_	K-30 Probe (CO_2_ meter)	*80 × 60 × 30 mm*	*Non Dispersive Infrared (NDIR)*	n.a	n.a
[42]	Gall et al., 2016	CO_2_	CM-0018 (CO_2_ Meter)	*146 × 91 × 33 mm*	*Non Dispersive Infrared (NDIR)*	*0–10,000 ppm*	n.a
[39]	Guevara et al., 2012	CO	MQ-7 Carmon Monoxide Semiconductor	*16 mm (sensor diameter)*	*Semiconductor*	*10–10,000 ppm*	n.a
[43]	Hu et al., 2011	CO_2_	H-550 EV	*38 × 32 × 12 mm (sensor)*	*Non Dispersive Infrared (NDIR)*	0–5000 ppm	n.a
[44]	Kanjo et al., 2008	CO	n.a	n.a	n.a	n.a	n.a
[9]	Lo Re et al., 2014	O_3_	n.a	n.a	n.a	n.a	n.a
		CO					
		CO_2_					
		NO_2_					
[6]	Mead et al., 2013	CO	CO: CO-AF (Alphasense)	All sensors: 183 × 95 × 35 mm (445 g)	All sensors: Electrochemical	n.a	n.a
		NO	NO: NO-A1 (Alphasense)				
		NO_2_	NO_2_: NO_2_-A1 (Alphasense)				
[46]	Negi et al., 2011	Hydrocarbon and acid	n.a	Dimension comparable with a common smartphone (<250 g)	n.a	n.a	n.a
**PM and Gas Sensors**							
[31]	Hasenfratz et al., 2015	UFP	UFP: DiSCsMini (Matter Aerosol)	UFP: 40 × 90 × 180 mm (700 g)	*UFP: unipolar charger*	UFP: 10^3^–10^6^ particle/cm^3^	n.a
		O_3_	O_3_: MiCS-OZ-14 (e2v)	O_3_: n.a	O_3_: semiconductor	*O_3_: 20–200 ppb*	
		CO	CO: CO-B4 (Alphasense)	*CO: 32 mm(sensor’s diameter)*	CO: electrochemical	CO: n.a	
		NO_2_	NO_2_: NO_2_-B4 (Alphasense)	*NO_2_: 32 mm (sensor diameter)*	NO_2_: electrochemical	NO_2_: n.a	
[36]	Mueller et al., 2016	UFP	UFP: DiSCsMini (Matter Aerosol)	UFP: 40 × 90 × 180 mm	UFP: Unipolar diffusion charger	UFP: 10^3^–10^6^ particle/cm^3^	n.a
		O_3_	*O_3_: MiCS-OZ-14 (e2v)*	O_3_: n.a	*O_3_: electrochemical*	*O_3_: 20–200 ppb*	
		CO	*CO: CO-B4 (Alphasense)*	*CO: 32 mm (sensor diameter)*	*CO: electrochemical*	CO: n.a	
[5]	Pokrić et al., 2015	PM	PM: OPC-N1 (Alphasense)	*PM: n.a*	O_3_: electrochemical	O_3_: 0–2 ppm	n.a
		O_3_	O_3_: O_3_-B4 (Alphasense)	*O_3_: 32 mm*	CO: electrochemical	CO: 0–50 ppm	
		CO	CO: CO-B4 (Alphasense)	*CO: 32 mm*	CO_2_: infrared	CO_2_: 0–5000 ppm	
		CO_2_	CO_2_: CO_2_-IRC-AT (Alphasense)	*CO_2_: 20 mm*	NO: electrochemical	NO: 0–20 ppm	
		NO	NO: NO-B4 (Alphasense)	*NO: 32 mm*			
[3]	Velasco et al., 2016	PM_10_	GPY21010AU0F (Sharp)	*PM_10_: 46* × *30* × *17 mm*	PM_10_: Light scattering	PM_10_: 0–0.5 mg/m^3^	PM_10_: 5 V (0.1 mg/m^3^)
		O_3_	MiCS-2610 (e2v Technologies Ltd)	*O_3_: 9 mm*	O_3_: n.a	O_3_: 10–1000 ppb	O_3_: 2–4 ohm

**Table 4:** Presence or absence of GPS, wireless and web/smartphone application technologies in reported studies.

Study	GPS	Wireless	Application
**PM Sensors**			
Wong et al. [37]	Yes	Yes	Yes
**Gas Sensors**			
Al-Ali et al. [38]	Yes	Yes	Yes
Castell et al. [29]	Yes	Yes	Yes
Chen et al. [45]	Yes	Yes	Yes
Eisenman et al. [40]	Yes	Yes	Yes
Fu et al. [41]	Yes	Yes	Yes
Gall et al. [42]	No	No	No
Guevara et al. [39]	Yes	Yes	Yes
Hu et al. [43]	Yes	Yes	No
Kanjo et al. [44]	Yes	Yes	Yes
Lo Re et al. [9]	Yes	Yes	No
Mead et al. [6]	Yes	No	No
Negi et al. [46]	No	Yes	Yes
**PM and Gas Sensors**			
Hasenfratz et al. [31]	Yes	Yes	No
Mueller et al. [36]	Yes	No	No
Pokrić et al. [5]	Yes	Yes	Yes
Velasco et al. [3]	Yes	Yes	No

**Table 5:** Disadvantages and advantages related to the use of MMs.

**Disadvantages**
MMs seemed to be less accurate as compared with reference methods
Performance and reliability of MMs have yet to be fully evaluated
**Advantages**
MMs have the capability of providing continuous and real-time data
Data acquired via MMs are characterized by high spatial resolution
Data acquired via MMs are characterized by high temporal resolution
MMs can potentially be used in different application (indoor/outdoor air quality monitoring; community/individual exposure)
MMs can adapt to different experimental designs

## Supplementary Materials

The following are available online at www.mdpi.com/1660-4601/14/8/909/s1, Table S1: Studies’ characteristics. Data that were not directly acquired from the paper are reported in italics. In the case of missing data within the reference papers, data were acquired from the literature cited in bibliography or from external sources (retailer’s site), Table S2: Summary of selected papers. Aim of the study, methods and principal results are reported.
