# Supplementary material for: Miniaturized Monitors for Assessment of Exposure to Air Pollutants: A Review
Source: Int J Environ Res Public Health. 2017 Aug 12;14(8):909. doi: 10.3390/ijerph14080909 (PMC5580612; doi:10.3390/ijerph14080909)
Supplement: Supplementary file 1 [file ijerph-14-00909-s001.pdf]

Supplementary Materials: Miniaturized Monitors for Assessment of Exposure to Air Pollutants: A Review

Francesca Borghi, Andrea Spinazzè, Sabrina Rovelli, Davide Campagnolo, Luca Del Buono, Andrea Cattaneo and Domenico M. Cavallo

Table S1. Studies' characteristics. Data that were not directly acquired from the paper are reported in italics. In the case of missing data within the reference papers, data were acquired from the literature cited in bibliography or from external sources (retailer's site).

Reference	Study	Monitoring Site	Period	Pollutants	Mobile/Stationary	Indoor/Outdoor	Dimension and Weight	Wireless	GPS	Application	Power	Mesurement Range	Response Time
**PM sensors**													
[1]	Wong et al., 2014	Hong - Kong	apr-14	$PM_{2.5}$	Mobile	Indoor<br>Outdoor	113x89 mm	Bluetooth	Yes	Yes	30 hours	n.a	1 s
**Gas sensors**													
[2]	Al-Ali et al., 2010	Sharjah	n.a	CO	Mobile	Outdoor	n.a	Yes	Yes	Yes	n.a	CO: 0 - 1000 ppm	CO <25s
				NO_2								NO_2: 0 - 20 ppm	NO <60s
				SO_2								SO_2: 0 - 20 ppm	SO_2 <25s
[3]	Castell et al., 2015	Oslo	n.a	O_3	Mobile	Outdoor	*32 mm (sensor diameter)*	Yes	Yes	Yes	Platform will be mounted on an electrical bicycle and the power of the platform will be supplied from the bicycle battery	Concentrations typically found in urban environment	*O_3 <45s*
				CO									*CO <25s*
				CO_2									*CO_2 n.a*
				NO									*NO <45s*
				NO_2									*NO_2 <60s*
				SO_2									*SO_2 <30s*
[4]	Chen et al., 2012	Phoenix	n.a	VOC	Mobile	Indoor	Not much larger than a common smartphone	Yes	Yes	Yes	10 hours	Environmental: 0 - 1 ppm	Raw data: 1 s per measurement
		San Diego				Outdoor	(<300 g)					Industrial: 1 - 1000 ppm	Calibrated concentration: 3 min per data

													point
[5]	Eisenman et al., 2009	Hannover	August 2006 - August 2007	CO_2	Mobile	Outdoor	*150x70 mm*	Yes	Yes	Yes	*10 hours (4 AA batteries)*	*0-2500 ppm or 0-4000 ppm*	*< 60s*
[6]	Fu et al., 2012	Singapore	n.a	CO_2	Mobile<br>Stationary	Outdoor	*80x60x30 mm*	Yes	Yes	Yes	n.a	n.a	*Warm up time: <60s*
[7]	Gall et al., 2016	Singapore	May - December 2015	CO_2	Mobile	Indoor<br>Outdoor	*146x91x33 mm*	No	No	No	*AA Batteries*	*0-10000 ppm*	1 min
[8]	Guevara et al., 2012	Asuncion	n.a	CO	Mobile	Outdoor	*16 mm (sensor diameter)*	Yes	Yes	Yes	n.a	*10-10000 ppm*	n.a
[9]	Hu et al., 2011	Hsinchu	n.a	CO_2	Mobile	Indoor<br>Outdoor	*38x32x12 mm*	Yes	Yes	No	n.a	0-5000 ppm	30 s
[10]	Kanjo et al., 2008	Bristol	n.a	CO<br>Noise	Mobile	Indoor<br>Outdoor	n.a	Bluetooth	Yes	Yes	n.a	n.a	n.a
[11]	Lo Re et al., 2014	Palermo	n.a	O_3<br>CO<br>CO_2<br>NO_2	Mobile	Outdoor	n.a	Yes	Yes	No	n.a	n.a	n.a
[12]	Mead et al., 2013	Cambridge<br>London<br>Cranfield<br>Valencia<br>Kuala Lampur<br>Lagos	2010	CO<br>NO<br>NO_2	Mobile<br>Stationary	Outdoor	183x95x35 mm	No	Yes	No	14 hours	n.a	n.a
[13]	Negi et al., 2011	n.a	n.a	Hydrocarbons and acid	Mobile	Indoor<br>Outdoor	Size comparable with a smart cell phone (<250 g)	Yes<br>Bluetooth	No	Yes	9h	n.a	n.a
						**PM and gas sensors**							
[14]	Hasenfratz et al., 2015	Zurich	April 2012 - April 2014	UFP<br>O_3<br>CO<br>NO_2	Mobile	Outdoor	UFP: 40x90x180 mm (700g)<br>O_3: n.a<br>*CO: 32 mm (sensor diameter)*<br>*NO_2: 32 mm (sensor diameter)*	Yes	Yes	No	UFP: 20 hours	UFP: 10^3 - 10^6 particles/ccm	n.a

[15]	Mueller et al., 2016	Zurich	July - September 2013	UFP	Mobile	Outdoor	UFP: 4x9x18 cm (700g)	No	Yes	No	*UFP: 20 h*	UFP: 10^3 - 10^6 particles/ccm	n.a
			December 2013 - February 2014	O_3	Stationary		O_3: n.a						
				CO			*CO: 32 mm (sensor diameter)*						
[16]	Pokrić et al., 2015	n.a	n.a	PM_1			n.a	Yes	Yes	Yes	n.a	O_3: 0 - 2 ppm	n.a
				$PM_{2.5}$								CO: 0 - 50 ppm	
				PM_{10}	Mobile	Indoor						CO_2: 0 - 5000 ppm	
				O_3	Stationary	Outdoor						NO: 0 - 20 ppm	
				CO								NO_2: 0 - 20 ppm	
				CO_2									
				NO									
				NO_2									
[17]	Velasco et al., 2016	Turin	n.a	PM_{10}	Mobile	Outdoor	*PM_{10}: 46x30x17 mm*	Yes	Yes	No	n.a	PM_{10}: 0 - 0.5 mg/m^3	30 s
				O_3			*O_3: 9mm*					O_3: 10 - 1000 ppb	

Table S2. Summary of selected papers. Aim of the study, methods and principal results are reported.

Reference	Study	Aim of the Study	Methods	Principal Results
		**PM sensors**		
[1]	Wong et al., 2014	Development and evaluation of an Integrated Environmental Monitoring System.	Field test conducted in different environments and locations both outdoor (e.g. road repair work, bus stop) and indoor.	The system illustrated worked well during field test and provide an important platform, raising the public awareness regarding environmental quality in micro environments. GPS performance are related to the number and the position of GPS satellites.
		**Gas sensors**		
[2]	Al-Ali et al., 2010	Design, implementation and test of a wireless distributed mobile air pollution monitoring system.	The system was placed on a university bus.	Data transmitted and available on Internet shows pollution level and their conformance to local air quality standards.
[3]	Castell et al., 2015	Present a new approach for the development of information chain.	Measurement were conducted outdoor, using mobile vehicles (e.g. bicycles, bus).	Main results not reported but authors, demonstrating the feasibility of mobile sensor network explains how this kind of data can contribute to understanding about air quality issue.

[4]	Chen et al., 2012	Present a wearable sensor able to provide improvement in understanding of indoor and outdoor personal exposure assessment to VOC. Validation of VOC sensor in real-word environments and in different scenarios.	Validation of VOC sensor's performance using Gas Chromatography and Selected Ion Flow Tube e mass Spectrometry reference Methods in different environments (indoor/outdoor, traffic…) and scenarios (e.g. different work, paint remodeling…).<br><br>Calibration and validation of the VOC sensor in real-word environments.<br><br>Different tests were conducted: fields tests, inter laboratory tests and validation with commercial instruments.	Field test validated the instrument's performance.<br><br>Field test demonstrate high temporal and spatial pollutants information provided by this innovative monitor.<br><br>The sensor correlates well or outperforms similar VOC sensor.<br><br>The sensor may be able to improve knowledge about personal exposure, protecting human health.<br><br>The sensor provide an accuracy higher than 81%.
[5]	Eisenman et al., 2009	Authors presents design, implementation and evaluation of a mobile sensing system.	Reported implementation were tested during bicycle trips on different routes.	The study reported is the first to quantify cyclist experience (personal performance and shared sampling).
[6]	Fu et al., 2012	Reports design of environmental monitoring system and related critical issue.	Light-weight and low-cost sensors were installed on mobile vehicles (e.g. cars, scooters, bicycle) and placed in outdoor fixed stations.	Three critical issues related design of environmental monitoring have been reported: efficiency of sensors, coverage of monitoring area, validity of sensed data.
[7]	Gall et al., 2016	An exposure study was conducted, in order to understand levels, dynamics and influencing factors of personal exposure to CO_2.	Personal, portable and continuous monitoring characterized by high time resolution were used for indoor and outdoor campaign.	Some determinant of CO_2 exposure were found.
[8]	Guevara et al., 2014	Describes design and implementation of an environmental wireless sensor network.<br><br>The study reports the development of	Sensor network was tested following selected routes of public transports.	Environmental monitors was characterized by good performance.

		the sensor network and results obtained.		
[9]	Hu et al., 2011	Propose a vehicular sensor network architecture.<br><br>Development of a Zig-Bee based prototype in order to monitor carbon dioxide in urban areas.	Measurement conducted outdoor and indoor, via vehicular sensor node (characterized by internal and external sensors).	Reported results are related to simulation data and prototype experience.
[10]	Kanjo et al., 2008	Describes a monitor system able to monitors using mobile phones.	Evaluation of sensor in a school environment.	Authors demonstrate the feasibility of developing an environmental sensing monitors using mobile sensing devices.
[11]	Lo Re et al., 2014	Show and discuss different approach to environmental monitoring.	Use of mobile monitor device as implementation of vehicular sensor network.<br><br>Monitors used outdoor on public busses.	Vehicular sensor network is an innovative approach to environmental monitoring and it is considered as an interesting development in wireless and mobile networking.<br><br>The main advantage of this approach is relative to the economy and to the simplicity of the system.
[12]	Mead et al., 2013	Provide evidence about performance of electrochemical sensor network.	Measurement via portable devices held by pedestrian and cyclist/drivers and via static devices in outdoor.<br><br>Different test carried out in urban environments.<br><br>Laboratory test and validation carried out using standard gas.	The study showed that urban environment cannot fully characterized using static networks.<br><br>Networks characterized by high spatial and temporal resolution are required in urban air quality measurements.<br><br>In field measurement, the sensor baseline signal depends on environmental conditions (temperature and relative humidity).<br><br>The work demonstrate the potential of miniaturized and low-cost sensor network system utilized in urban environment and its ability to provide data at appropriate scale<br><br>Sensor operate without significant gain attenuation over long period.
[13]	Negi et al., 2011	Paper reports development and test of a wearable monitor.	Validation of function and performance in different scenarios, involving operator from different working fields.<br><br>Sensor validation was carried out with chromatography-mass	Monitors provide accurate and real-time measurement and it is immune to environmental changes (e.g. humidity, temperature…).

			spectrometer (GC-MS) and performances were compared with commercial instrument.	
**PM and gas sensors**				
[14]	Hasenfratz et al., 2015	Development of a land-use regression model in order to create pollution maps, characterized by high spatial resolution.	Measurement carried out on the top of public vehicles.	Accuracy of pollution maps (characterized by sub-weekly temporal resolution) suffers from the reduced number of measurement available.<br><br>Maps generate with this new approach are characterized by higher spatial and temporal resolution and are useful to general population as well environmental scientist and epidemiologist.
[15]	Mueller et al., 2016	Show performance of electrochemical sensor in urban environment.<br><br>Present a method to modelling pollutant concentrations in urban environments relying on georeferred data acquired via mobile sensor network.	Measurements were carried out both with stationary and mobile devices.<br><br>Mobile monitors were installed on the roof of public transports.<br><br>Model validation with data from fixed stations.	Sensor used in monitor test showed low noise and high linearity.<br><br>The modelling approach proposed reasonably predict the main features of the investigated pollutant.
[16]	Pokrić et al., 2015	Describes an approach to integration of physical and digital worlds.<br><br>Demonstrate how Internet of Things (IoT) and Augmented Reality (AR) could provide a new way to present and share digital information in real world.	Different kind of application in real-word (stationary/mobile and indoor/outdoor).	Several users find that this new approach promotes environmental issue and that the game proposed was educational an entertaining.
[17]	Velasco et al., 2016	The paper describes a mobile wireless sensor network with the aim to complement existing air quality	PM_{10} and O_3 sensors were tested in controlled environments (outdoor/indoor) and during different on-field campaigns (urban and rural locations), principally using	This system, due to its high portability, may be able to improve spatial resolution and resolution of data.<br><br>Test conducted on field showed that sensors are able to provide accurate data, under adequate calibration and maintenance conditions.

	monitoring system.	bike sharing support and static stations.	Measurement conducted via proposed sensor were less accurate than reference methods.

References

1. Wong, M.S.; Yip, T.P.; Mok, E. Development of a personal integrated environmental monitoring system. *Sensor* **2014**, doi:10.3390/s141122065.
2. Al-Ali, A.R.; Zualkernan, I.; Aloul, F. A mobile GPRS-sensor array for air pollution monitoring. *Sensors* **2010**, doi:10.1109/JSEN.2010.2045890.
3. Castell, N.; Kobernus, M.; Liu, H.Y.; Schneider, P.; Lahoz, W.; Berre, A.J.; Noll, J. Mobile technologies and services for environmental monitoring: The citi-sense-mob approach. *Urban Clim.* **2015**, doi:10.1016/j.uclim.2014.08.002.
4. Chen, C.; Driggs Campbell, K.; Negi, I.; Iglesias, R.A.; Owens, P.; Tao, N.; Tsow, F.; Forzani, E.S. A new sensor for the assessment of personal exposure to volatile organic compounds. *Atmos. Environ.* **2012**, doi:10.1016/j.atmosenv.2012.01.048.
5. Eisenman, S.B.; Miluzzo, E.; Lane, N.D.; Peterson, R.A.; Ahn, G.; Campbell, A.T. BikeNet: A mobile sensing system for cyclist experience mapping. *ACM Trans. Sens. Netw.* **2009**, doi:10.1145/1653760.1653766.
6. Fu, H.; Chen, H.; Lin, P. APS: Distributed air pollution sensing system on wireless sensor and robot networks. *Comput. Commun*. **2012**, doi:10.1016/j.comcom.2011.08.004.
7. Gall, E.T.; Cheung, T.; Luhung, I.; Schiavon, S.; Nazaroff, W.W. Real-time monitoring of personal exposures to carbon dioxide. *Build. Environ.* **2016**, doi:10.1016/j.buildenv.2016.04.021.
8. Guevara, J.; Barrero, F.; Vargas, E.; Becerra, J.; Toral, S. Environmental wireless sensor network for road traffic applications. *IET Intell. Trans. Syst.* **2012**, doi:10.1049/iet-its.2010.0205.
9. Hu, S.; Wang, Y.; Huang, C.; Tseng, Y. Measuring air quality in city areas by vehicular wireless sensor networks. *J. Syst. Softw.* **2011**, doi:10.1016/j.jss.2011.06.043.
10. Kanjo, E.; Benford, S.; Paxton, M.; Chamberlain, A.; Stanton Fraser, D.; Woodgate, D.; Crellin, D.; Woolard, A. MobGeoSen: Facilitating personal geosensor data collection and visualization using mobile phones. *Pers. Ubiquit. Comput.* **2008**, doi:10.1007/s00779-007-0180-1.
11. Lo Re, G.; Perri, D.; Vassallo, S.D. Urban air quality monitoring using vehicular sensor networks. *Adv. Intel. Syst. Comput.* **2014**, *2063*, 311–232.
12. Mead, M.I.; Popoola, O.A.M.; Stewart, G.B.; Landshoff, P.; Calleja, M.; Hayes, M.; Baldovi, J.J.; McLeod, M.W.; Hodgson, T.F.; Dicks, J.; et al. The use of electrochemical sensors for monitoring urban air quality in low-cost, high-density networks. *Atmos. Environ.* **2013**, *70*, 186–203.
13. Negi, I.; Tsow, F.; Tanwar, K.; Zhang, L.; Iglesias, R.A.; Chen, C.; Rai, A.; Forzani, E.S.; Tao, N. Novel monitor paradigm for real-time exposure assessment. *J. Expo. Sci. Environ. Epidemio.* **2011**, doi:10.1038/jes.2010.35.
14. Hasenfratz, D.; Saukh, O.; Walser, C.; Hueglin, C.; Fierz, M.; Arn, T.; Beutel, J.; Thiele, L. Deriving high-resolution urban air pollution maps using mobile sensor nodes. *Pervasive Mob. Comput.* **2015**, doi:10.1016/j.pmcj.2014.11.008.
15. Mueller, M.D.; Hasenfratz, D.; Saukh, O.; Fierz, M.; Hueglin, C. Statistical modelling of particle number concentration in Zurich at high spatio-temporal resolution utilizing data from a mobile sensor network. *Atmos. Environ.* **2016**, doi:10.1016/j.atmosenv.2015.11.033.
16. Pokrić, B.; Krčo, S.; Drajić, D.; Pokrić, M.; Rajs, V.; Mihajlović, Z.; Knežević, P.; Jovanović, D. Augmented reality enabled IoT services for environmental monitoring utilising serious gaming concept. *J. Wirel. Mobile Netw.* **2015**, *6*, 37–55.
17. Velasco, A.; Ferrero, R.; Gandino, F.; Montrucchio, B.; Rebaudengo, M. A mobile and low-cost system for environmental monitoring: A case study. *Sensor* **2016**, doi:10.3390/s16050710.

© 2017 by the authors; licensee MDPI, Basel, Switzerland. This article is an open access article distributed under the terms and conditions of the Creative Commons by Attribution (CC-BY) license (http://creativecommons.org/licenses/by/4.0/).
